# Investigation of Recycled and Coextruded PLA Filament for Additive Manufacturing

**DOI:** 10.3390/polym14122407

**Published:** 2022-06-14

**Authors:** Jana Sasse, Lukas Pelzer, Malte Schön, Tala Ghaddar, Christian Hopmann

**Affiliations:** Institute for Plastics Processing, RWTH Aachen University, 52074 Aachen, Germany; lukas.pelzer@ikv.rwth-aachen.de (L.P.); malte.schoen@ikv.rwth-aachen.de (M.S.); tala.ghaddar@alumni.fh-aachen.de (T.G.); office@ikv.rwth-aachen.de (C.H.)

**Keywords:** additive manufacturing, coextrusion, polylactide acid, recycling, filament

## Abstract

Polylactide acid (PLA) is one of the most used plastics in extrusion-based additive manufacturing (AM). Although it is bio-based and in theory biodegradable, its recyclability for fused filament fabrication (FFF) is limited due to material degradation. To better understand the material’s recyclability, blends with different contents of recycled PLA (rPLA) are investigated alongside a coextruded filament comprised of a core layer with high rPLA content and a skin layer from virgin PLA. The goal was to determine whether this coextrusion approach is more efficient than blending rPLA with virgin PLA. Different filaments were extruded and subsequently used to manufacture samples using FFF. While the strength of the individual strands did not decrease significantly, layer adhesion decreased by up to 67%. The coextruded filament was found to be more brittle than its monoextruded counterparts. Additionally, no continuous weld line could be formed between the layers of coextruded material, leading to a decreased tensile strength. However, the coextruded filament proved to be able to save on master batch and colorants, as the outer layer of the filament has the most impact on the part’s coloring. Therefore, switching to a coextruded filament could provide economical savings on master batch material.

## 1. Introduction

With benefits such as tool-less material processing, high geometric freedom, fast prototyping and cost-efficient small-scale production, additive manufacturing (AM) has the potential to revolutionize the manufacturing industry. This is reflected in the current value of the AM market, which is estimated at USD 12.6 billion in 2020 with a 21% year over year growth. The current projection estimates the AM market to reach a value of USD 37.2 billion in 2026 [1]. Material extrusion technologies, accounting for the largest share of the AM market [2], are expected to grow even faster at a rate of 27.43% between 2018 and 2024 [3]. To sustain such growth, users are demanding more sustainable technologies and materials [4]. This is particularly apparent when considering the plastics industry. With a global polymer use of 300 Mt in 2019 and an estimated 350 Mt in 2023, the consumption of resources is at an all-time high [5].

While the increase in extrusion-based systems, such as fused filament fabrication (FFF), also increases material use and waste, i.e., through failed parts or support structures [6], it can have a substancial impact on creating a more sustainable manufacturing environment. Based on the technology’s high degree of freedom when creating parts, internal structures can be filled sparsely, resulting in reduced material use and lightweight parts [7,8]. Because volumetric elements are added rather than subtracted, material utilization is high [6]. AM also has the potential to avoid over-production by manufacturing on demand [7,8]. Finally, since extrusion-based systems process thermoplastics, excess material can be recycled [9]. At this point, however, an endless closed loop of reusing the same material indefinitely is not possible [10].

The durability of polylactide acid (PLA), one of the most used polymers in extrusion-based AM [11], is limited, as PLA degrades over time and with every processing step. Limiting factors on the recyclability of PLA include thermal decomposition, hydrolysis, photo-oxidation, natural weathering and thermo-oxidative degradation [12]. While hydrolytic degradation can be part of its desired properties as it is key to PLA’s biodegradability, it can also limit the applications for this material. Thermal decomposition and thermo-oxidative degradation are the most dominant factors regarding recyclability. With each cycle of additive manufacturing, shredding and the production of new filament from PLA, the material is re-extruded at high temperatures, leading to random chain scission responsible for a reduction in molar mass, which in turn affects the glass transition temperature $T_{g}$ and the degree of crystallinity [12]. Previous research has shown that PLA displays brittle behavior when the molar mass ${\bar{M}}_{n}$ drops below 40 kg/mol [12]. Amorin et al. [13] have shown a reduction in molar mass from an initial ${\bar{M}}_{n}=70.7$ to ${\bar{M}}_{n}=61.7$ and an increase of the melt flow index (MFI) of about 57.6% after five extrusion cycles.

These accumulated degradation mechanisms lead to decreased mechanical properties of recycled PLA (rPLA) compared to virgin PLA, which can be measured, e.g., using injection-molded samples of both virgin and recycled PLA. Ženkiewicz et al. [14] found a reduction in tensile strength by 5.2% and a reduction in impact strength by 20.02% after ten extrusion cycles. Another study conducted by Budin et al. [15] found a decrease in tensile strength by 11%, along with a 5% decrease in transverse rupture strength, a 50% decrease in impact strength and a 4% decrease in hardness for the rPLA samples.

In addition to the mechanical degradation mechanisms, the process of additive manufacturing introduces new challenges for the use of rPLA due to the delicacy of the filament and additional requirements on the weld lines between layers. An investigation by Anderson [16] analyzed the mechanical properties of additively manufactured samples using both virgin and recycled PLA and a decrease in tensile strength by 10.9%, an increase in shear strength by 6.8% and a decrease in hardness by 2.4% were found, with increased variability in the results and occasional nozzle blockage with the recycled filament. An analysis by Cruz et al. [17] found no significant decrease in tensile strength at break across five reprocessing cycles, although a reduction in the strain at break of 10.63% was observed. In addition, a decrease in molecular weight by 46.91% after five reprocessing cycles and a six-fold increase in the MFI were found. Breški et al. [18] conducted a study investigating the suitability of recycled PLA filaments for additive manufacturing processes. The authors found inconsistent filament diameters and subsequent potential nozzle blockage for filament made from recycled PLA. Another study conducted by Babagowda et al. [19] investigating the mechanical properties of blends with virgin and recycled PLA found that apart from the recycled PLA content, the layer thickness in the printed test samples also played an important role in the tensile and flexural strength.

A review by Pakkanen et al. [20] found that while relevant, the field of recycling PLA for additive manufacturing purposes is still insufficiently studied. The authors suggest the use of a blend of virgin and recycled material to find an acceptable trade-off between mechanical properties and environmental concerns. They also point out that improvements in waste management are critical for this endeavor, as the contamination of material from post-consumer waste can pose a problem for the recyclability of PLA. A review by Shanmugam et al. [21] found that existing research to justify the use of recycled plastics in additive manufacturing was still lacking with regard to bending characteristics, the influence of FFF process parameters and the bonding between the layers of printed recycled parts.

The weakened mechanical properties of additively manufactured parts produced using rPLA largely stem from the weakened weld line between layers. Due to the laminar flow of the heated filament in the hotend, the material on the outer layer of the filament is also the material comprising the weld lines and outer layer visible to the consumer [22,23]. Therefore, the ‘ideal filament’ would have an outer layer with good optical properties and the capability to form strong weld lines, while the inner core of the filament could be comprised of recycled material in order to save cost and improve the ecological impact. While this method has already been used in other applications in plastics extrusion since the 1990s [24] and has applications in various fields such as profile extrusion [25], blow molding [26] and in food packaging [27], it recently has gained traction due to an increase in the demand of more sustainable packaging.

Recent research has already shown the potential of coextrusion in the production of filament for additive manufacturing applications. Ruckdashel at al. [23] have demonstrated how coextrusion can be used to enhance the content of carbon-based or inorganic fillers in 3D filament without compromising its mechanical properties. In another paper by Hart et al. [28], a method for the coextrusion of dual material filament was presented, where a star-shaped polycarbonate (PC) core is used to enhance the mechanical properties of acrylonitrile butadiene styrene (ABS) filament. However, to the authors’ knowledge, coextrusion has not been investigated as a tool to aid the processing of rPLA.

In this study, PLA filament is manufactured using varying proportions of recycled material, both in a monoextrusion and for the first time a coextrusion process. The parts manufactured from this filament are tested mechanically and optically to investigate the influence of various recycling strategies on part quality. The authors’ work intends to verify that the well-known benefits of coextrusion in the processing of recycled materials also apply to FFF. More specifically, the authors hypothesize that coextrusion gives better mechanical properties compared to simply blending virgin and recycled plastics, and that coextrusion can save on master batch. The work presented is significant due to its implications regarding the sustainability in plastics production by significantly reducing use of virgin material while maintaining high part quality. This contributes to the increasingly relevant fields of AM as well as sustainable practices in production by finding ways to incorporate recycled materials in existing processes.

## 2. Materials and Methods

All materials used were originally purchased in filament form and manually shredded to granulate size. As virgin material, PLA Extrafill Natural (Fillamentum Manufacturing Czech s.r.o., Hulin, Czech Republic) with 5% master batch material in the form of 3DJake EcoPLA White (Niceshops GmbH, Paldau, Austria) was used. The recycled material was produced from aged samples of PLA Neutral (German Reprap GmbH, Feldkirchen, Germany) mixed with 5% CCTree ST-PLA Pro Black (CCTree, Chenzhen, China) as a master batch material.

A rheological characterization of both materials was performed via plate–plate rheometry with a gap of 1 mm, a frequency of 1 Hz and an amplitude of 0.1% at 270 °C. The molecular weight can be deferred using Relation (1) described in [29].

(1) $$\eta_{0}=K\cdot M_{W}^{3.7},\;with\;lgK=-16.1$$

For the application of Relation (1), which is valid at 180 °C, a temperature correction using a WLF approach was used, with a glass transition temperature of $T_{g}=332.65\;K$[14], resulting in a standard temperature $T_{s}=382.65\;K$.

### 2.1. Extrusion

Prior to extrusion, all materials were dried in a dry air dryer at a temperature of 60 °C for two hours.

Extrusion trials were carried out using single-screw extruders with a 19 mm screw diameter and a length of 25 D. For monoextrusion, the screw was revolving at 15 rpm, corresponding to a throughput of 0.29 kg/h, and the temperatures in the barrel zones were set up as a rising temperature profile (170 °C, 180 °C, 190 °C) with a nominal temperature of 145 °C in the extrusion die. Downstream of the die, the haul off of a 3 m hot air shock canal was used as a cooling section for the extrudate with subsequent manual spooling. The haul-off speed was adjusted until a consistent filament diameter of 1.75 mm was reached and ranged from 1.5 to 1.8 m/min, depending on the rPLA content.

First, monoextruded filaments with varying degrees of rPLA content were produced. Continuous extrusion of filament was only achieved for material mixtures with up to 60% rPLA content. At proportions exceeding 60% rPLA content, the filament produced was too brittle for further processing. Therefore, monoextrusion filament samples were limited to 0%, 20%, 40% and 60% rPLA content, respectively. For the extrusion of 60% rPLA filament, the temperatures of the barrel zones on the extruder had to be reduced by 10 °C to achieve good strechability. With increasing rPLA content, higher variations in the filament diameter were observed, with diameters ranging between 1.6 and 1.8 mm.

**Figure 1:**
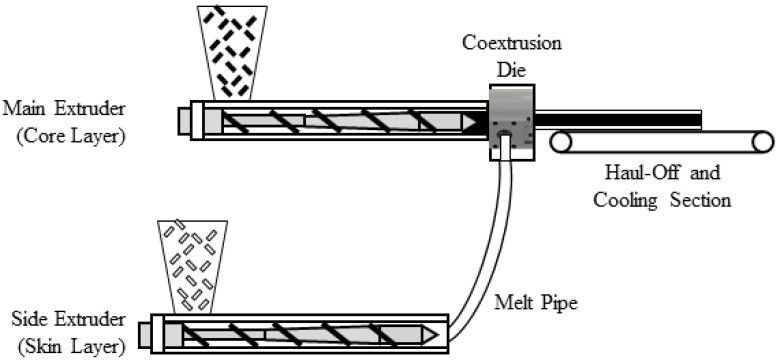
Schematic extrusion line configuration for coextrusion of PLA filament.

**Figure 2:**
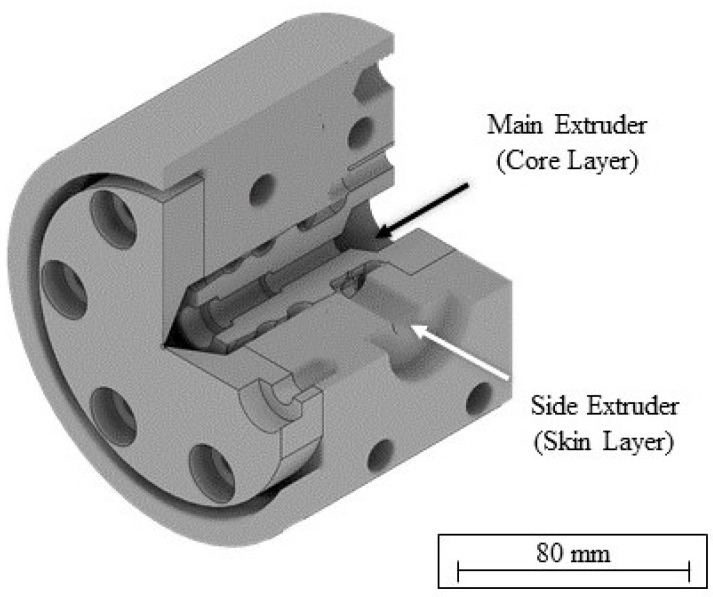
Coextrusion die based on a spiral mandrel die.

The coextruded filament was produced in an extrusion line setup visualized in Figure 1. In this setup, two single-screw extruders were used to feed the coextrusion die with both a core layer of blended material and a skin layer of virgin material. The coextrusion die (Figure 2) is based on a spiral mandrel die design for the skin layer, which is coating the core layer fed through the middle section. The coextruded filament was produced with a core layer with 60% rPLA content and a skin layer of virgin PLA. The ratio of the core and skin layer was controlled by the respective throughputs of the two extruders. For a 20% skin layer, the main extruder was set to 8 rpm, while the side extruder revolved at 2 rpm. This resulted in an overall rPLA content of 48% for the coextruded filament.

### 2.2. Additive Manufacturing

To evaluate the effect of using recycled material in filament production on the mechanical properties of manufactured parts, tensile tests and impact tests are conducted. The tensile tests are carried out according to DIN EN ISO 527. However, the test specimen geometry is adapted from the typically used 1BA sample and modified to be better suited to the FFF process. Specifically, the testing zone’s width is increased to 8 mm while the thickness is increased to 6 mm, allowing for enough volume to also include sparse infill. Additionally, the total length is increased to 90 mm while the testing zone’s length is decreased to 20 mm, allowing for a larger radius in the transition between the testing zone and the clamping zone. This decreases the chance for failure of the test specimen outside the testing zone, resulting in a noticeably increased number of valid tests. The charpy impact tests are conducted according to DIN EN ISO 791, with test specimen dimensions of 80 mm by 10 mm by 4 mm and a v-shaped notch, which is included in the specimen design and therefore manufactured during the AM process.

**Figure 3:**
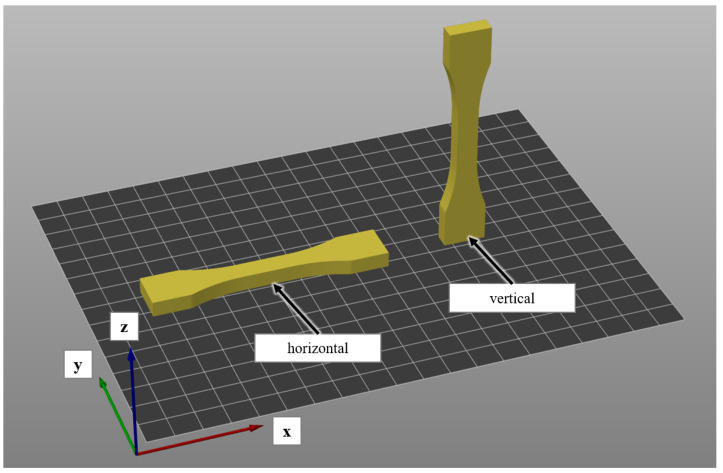
Horizontal manufacturing orientation (**left**) and vertical manufacturing orientation (**right**).

To evaluate the impact of various amounts of recycled filament on the strength of individual strands as well as inter-layer adhesion, all test specimens are manufactured in a horizontal and a vertical orientation (Figure 3). For this, a CR-10S 3D-printer is used. The machine is equipped with a 0.6 mm nozzle to prevent clogging from potential impurities in the filament. The G-Code is prepared using Slic3r version 1.3.0, which is an open source slicing software. For both build orientations, five specimens are manufactured from each monoextruded material (100% virgin PLA, 20% rPLA, 40% rPLA, 60% rPLA) and the coextruded material (48% rPLA). Additionally, five reference samples for both orientations are manufactured from virgin PLA filament. All additional process parameters are kept constant according to Table 1.

**Figure 4:**
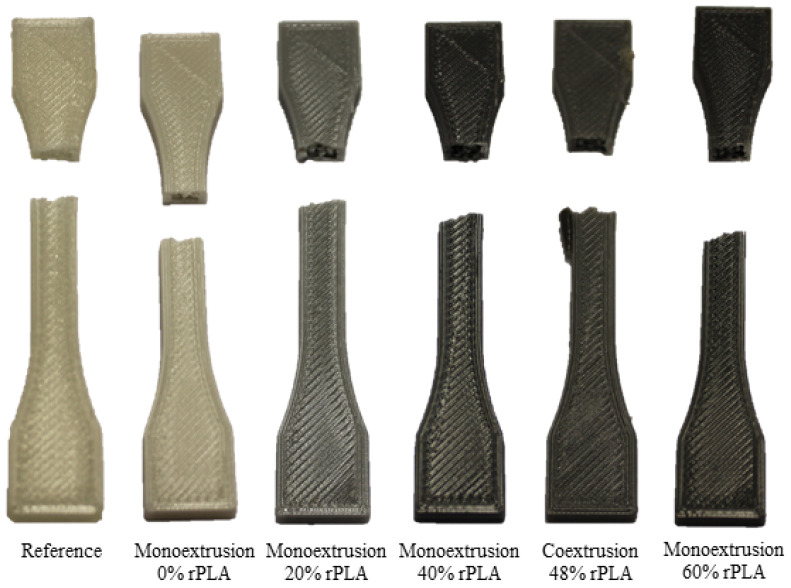
Tensile testing samples manufactured in horizontal position.

Figure 4 shows one specimen from each category after testing. While the reference sample is somewhat translucent, based on the natural, uncolored reference material, the samples from recycled material appear in various shades of gray, which are caused by the varying mixtures of white-colored virgin PLA and black-colored rPLA used during filament production.

## 3. Results

In the initial rheological characterization using plate–plate rheometry, the zero shear viscosity at 270 °C of the material with 100% virgin PLA was measured at 358.265 Pa s, while the zero shear viscosity at 270 °C of the recycled material was reduced by 71.7% with 101.322 Pa s. Using Relation (1), the molecular mass of the recycled material was approximated to be only 70.6% of the virgin material.

### 3.1. Mechanical Analysis

The manufactured tensile specimens were tested using a Z150 universal test machine which is equipped with a contact multiXtens extensometer for strain measurement. To ensure a constant load on the specimen in the clamping, the jaws were tightened to 15 Nm. Young’s modulus was measured at a speed of 1 mm/min, while the remaining test was conducted at 5 mm/min. The impact specimens were tested using a small impact pendulum Nr. 5102 with a 0.5 J impact hammer and a pendulum length of 0.225 m. All tests were conducted at room temperature.

The tensile strength tests were conducted with samples manufactured in both the horizontal and vertical position. While the tensile strength of the samples printed in the horizontal position mainly indicates the strength of the individual strands, the tensile strength of the samples printed in the vertical position is a measure for the strength of the weld line between the layers. For each material and printing orientation, five samples were analyzed, and ANOVA was performed to indicate the significance of the observed differences (p<0.05).

In the results section, all significant differences are marked in the diagrams using a Tukey’s honestly significant difference (HSD) test.

**Figure 5:**
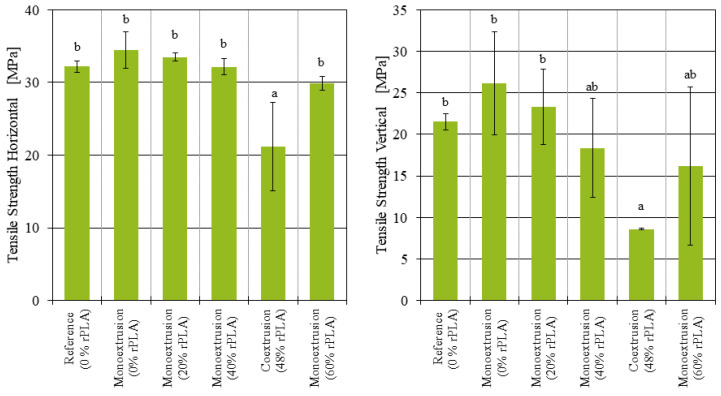
Tensile strength of additively manufactured samples manufactured in horizontal and vertical position.

The tensile strength of the monoextruded filament decreases with increasing rPLA content. Figure 5, on the left, shows the tensile strength of the samples manufactured in the horizontal position. The filament with 100% virgin PLA content had an average tensile strength of 34.45 MPa, and while it decreased with increasing rPLA content, the differences were not significant. The coextruded filament, on the other hand, was brittle, and the tensile strength was significantly lower than for all the monoextruded filaments. With an average of 21.17 MPa, the tensile strength was reduced by about 39% compared to the 100% virgin PLA filament.

The tensile strength of the specimens printed in the vertical position (Figure 5 right) was more inconsistent. Since a small defect or inconsistency in one layer is enough to initiate part failure, and the number of layers in vertical specimens is much larger than in horizontal specimens, while the area of each layer is much smaller for vertical samples, a larger variation in measured results is to be expected. With increasing rPLA content, the number of failed tests due to failure in the clamping zone also increased. As a result, only two out of five specimens at 60% rPLA content could be correctly analyzed. The two remaining specimens display divergent behavior, where one specimen’s tensile strength was measured at 6.63 MPa, while the other one’s was measured at 25.69 MPa. This makes an interpretation of the results difficult. For the vertical samples, the tensile strength of the monoextruded filaments decreased with increasing rPLA content. With 8.61 MPa, the tensile strength of the coextruded samples in the vertical position was reduced by 67% compared to the 100% virgin PLA content samples. However, only the difference in tensile strength between the samples with 100% virgin PLA content and the coextruded samples was found to be statistically significant (p=0.024). There was barely any variation in the tensile strength of the coextruded samples, and there were less failed tensile tests. This might suggest that the coextruded filament produced more reliable weld lines than the monoextruded filaments with 40% and 60% rPLA content.

**Figure 6:**
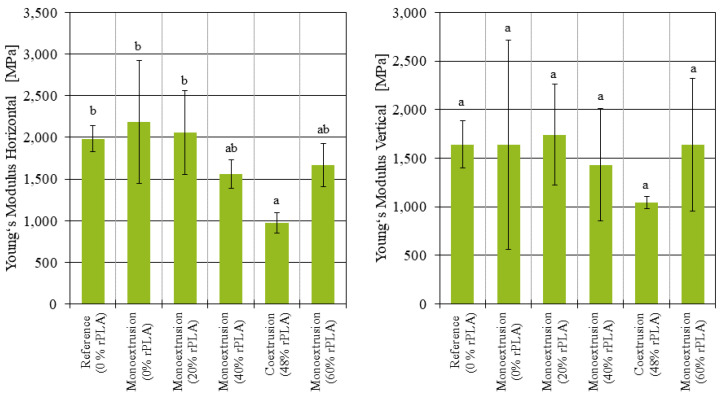
Young’s modulus of additively manufactured samples manufactured in horizontal and vertical position.

In addition, the Young’s modulus of the samples was evaluated. For the samples produced from monoextruded filament and printed in the horizontal position (Figure 6 left), no significant changes were observed. On the other hand, the Young’s modulus of the coextruded samples printed in the horizontal position was reduced by 55% compared to the samples with 100% virgin PLA (p=0.047). The results for the Young’s modulus of the samples printed in the vertical position are depicted in Figure 6 right. Here, no statistically significant differences between the monoextruded or coextruded samples were found.

**Figure 7:**
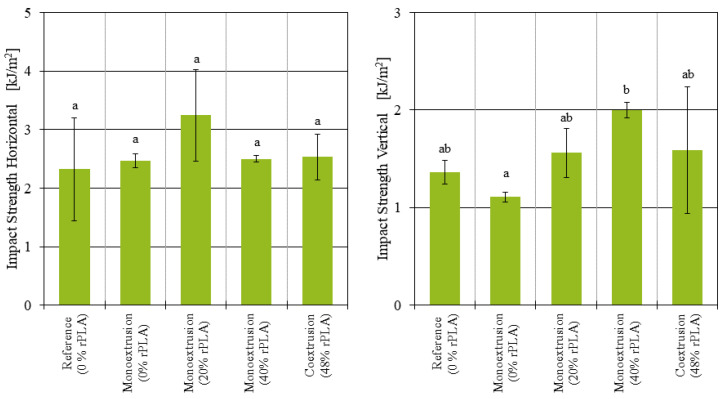
Charpy impact strength of additively manufactured samples manufactured in horizontal and vertical position.

The Charpy impact strength measured for the samples printed in the horizontal position (Figure 7 left) ranged between 2.32 and 3.24 kJ/m^2^, with no significant dependency from the rPLA content. The samples printed in the vertical position (Figure 7 right) had an impact strength between 1.11 and 2.00 kJ/m^2^. Here, the only difference found to be statistically significant (p=0.041) was between the samples printed from 100% virgin PLA filament and the samples printed from filament with 40% rPLA content.

### 3.2. Optical Analysis

To investigate a possible cause for the comparatively low mechanical performance of samples manufactured from coextruded filament and for the high standard deviation of some samples, optical analyses of the extruded filament and the manufactured test samples were performed.

**Figure 8:**
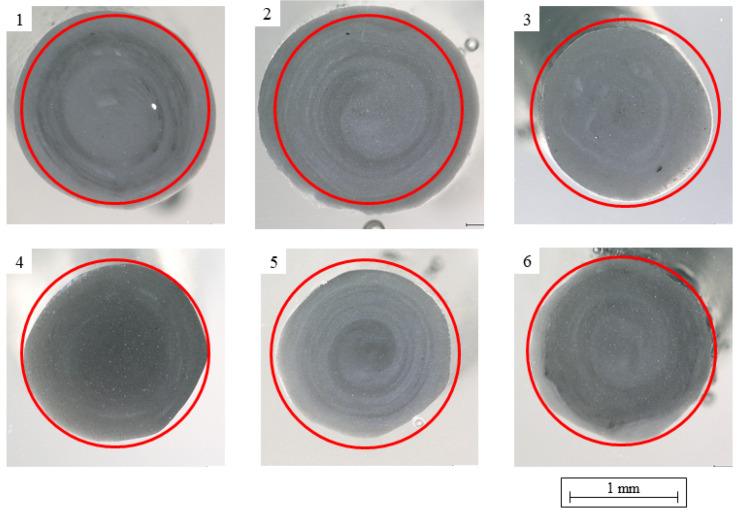
Microscopic analysis of six samples of the coextruded filament with a 20% skin layer, where the circle marks an ideal cross-section with d = 1.75 mm.

The coextruded filament was analyzed in bright-field microscopy as well as optical microscopy with differential interference contrast (DIC). A comparison of the cross-sections of different samples of the same coextruded filament shows high deviations from an idealized circular shape with a 1.75 mm diameter (Figure 8). While some coextruded samples were of roughly circular shape, others were much more inconsistent. Spiral patterns from the screw were apparent in all samples, suggesting a bad mixing behavior of material and master batch. Not all samples display clearly visible coextrusion with a clear interface between the core layer and skin layer. Sample a shows a clear interface between the two layers and is also of a roughly circular shape. Sample b, on the other hand, might show an interface, but the interface is much less pronounced. Samples c and d show no apparent signs of coextrusion. While sample e shows a roughly circular interface between the layers, the filament’s shape has a lot of kinks, suggesting an uneven extrusion of the skin layer. This is even more pronounced in sample f, where both the outer and the inner interface of the skin layer are noticeably uneven.

**Figure 9:**
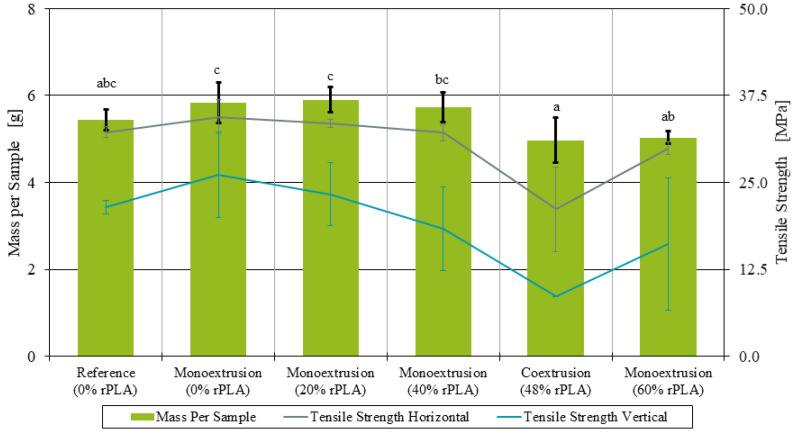
Average mass of tensile testing samples printed in both horizontal and vertical position.

One interpretation of these results might be that the skin layer was extruded at such a low throughput that no consistent material output was achieved. However, an increase in throughput was not feasible due to the limitations in haul-off speed and cooling line length in the lab setup used. In addition, many coextruded samples displayed a significantly smaller cross section area. This is also apparent in the mass of the printed samples, which is visualized alongside the tensile strengths in Figure 9. It was observed that both the tensile strength and the mass of the individual tensile testing specimens decreased with increasing rPLA content. The coextruded samples were on average 17% lighter than the 100% virgin PLA monoextruded samples ($p=1\cdot 10^{-4}$). The monoextruded filaments with 60% rPLA content show similar problems with respect to fluctuations in diameter and a 16% reduction in sample mass compared to the 100% virgin PLA samples (p=0.047). Since filament-based AM machines feed material based on length rather than based on weight or volume, every deviation in filament diameter and filament roundness influences the resulting parts. Based on the smaller filament diameter (Figure 8) and the lower measured weight of the samples, it can be concluded that less material than specified was introduced to the part. This, in turn, means that the layer cross-sections are smaller, resulting in lower mechanical properties when loaded in the strand direction. Additionally, layers are not compressed as much, and the interface between the layers is smaller, resulting in lower mechanical properties of the part when loaded in the build direction.

**Figure 10:**
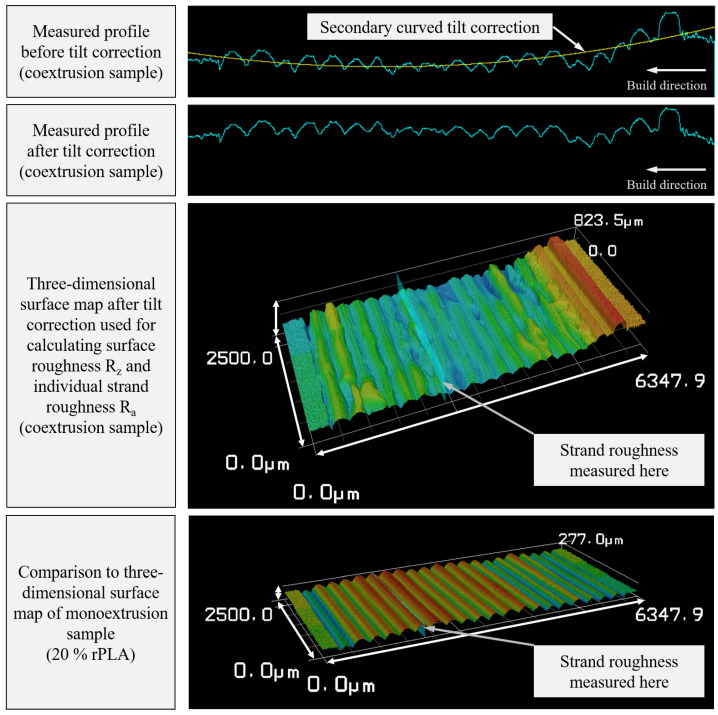
Surface tilt correction and three-dimensional surface map exemplified for the LSM measurement of a sample manufactured from coextruded filament. Every peak represents one individual layer.

Another aspect to consider is failure initiation due to the notch effect. To investigate whether the roundness deviation in the extruded filament results in increased roughness of the manufactured samples, an analysis of the surface roughness of the printed horizontal tensile testing samples was performed using a laser scanning microscope (LSM). This method enables the three-dimensional reconstruction of the part’s surface (Figure 10 bottom), allows for tilt and form correction (Figure 10 top), and calculates all relevant surface roughness metrics. To obtain an accurate comparison of the roughness caused by inconsistent filament diameter and roundness, the tilt and form correction was used to correct for bulging of the samples toward the heat bed. Known as elephant’s foot, this bulging is a typical artifact in FFF parts and can be a result of excess heat from the heat bed during the manufacturing process, preventing the lower layers from solidifying completely. As a result, those lower layers are compressed by the weight of subsequent layers. The bulging may also be attributed to an undersized gap between the nozzle and the bed during the first layer. By choosing a curved profile for correction, the bulge is accounted for, and surface roughness caused by the individual strands can be measured.

**Figure 11:**
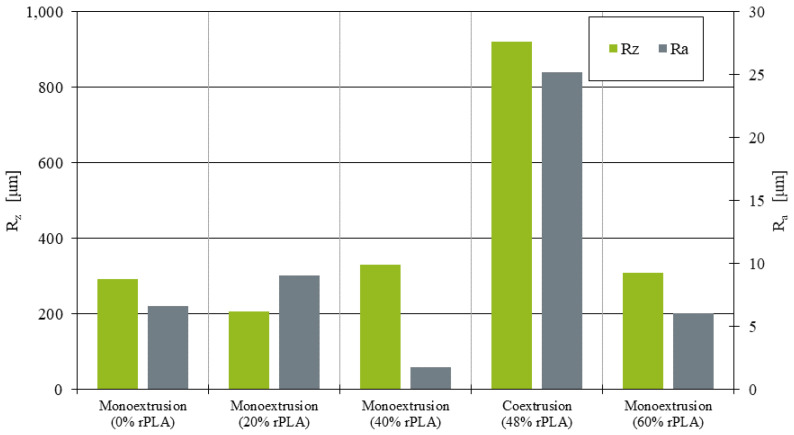
Surface roughness $R_{z}$ and individual strand roughness $R_{a}$ of additively manufactured tensile testing samples.

Using the corrected surfaces, the peak-to-valley height $R_{z}$ [30] over the sample’s complete depth was calculated. Figure 11 shows the distribution of roughness values for the parts manufactured from mono- and coextruded filaments. While the values for the monoextruded filament ranged from $R_{z}\;=\;206.62$ μm at 20% rPLA content to $R_{z}\;=\;328.47$ μm at 40% rPLA content, the surface roughness for the coextruded material was significantly higher at $R_{z}\;=\;921.01$ μm.

Similar results can be observed when evaluating single strands of material. Here, the arithmetic mean roughness value $R_{a}$ [30] was used to quantify deviation inside a single strand (right axis data in Figure 11). With values between $R_{a}\;=\;1.77$ μm at 40% rPLA content and $R_{a}\;=\;9.05$ μm at 20% rPLA content, individual strands manufactured from monoextruded filament showed only a low surface roughness. In comparison, the single-strand roughness of coextruded filament after the AM process was $R_{a}\;=\;25.17$ μm.

These investigations show the significantly higher roughness of the parts produced from coextruded filament, which can be attributed to the diameter and roundness deviation caused by the extrusion process. In addition to reducing the surface quality in manufactured parts, this can be a possible explanation for the coextruded sample’s lower mechanical performance.

The fluctuations in diameter of the filament with 40% and 60% rPLA content did not show in the surface roughness of the samples. On the other hand, the coextruded samples displayed a highly increased surface roughness, suggesting that the roundness of the filament cross-section is a much higher influence factor than fluctuations in the filament diameter over time.

**Figure 12:**
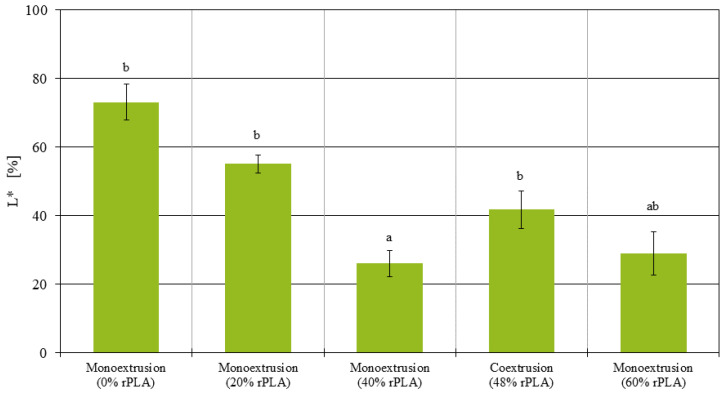
Reflectance of all additively manufactured tensile testing samples.

In addition, measurements of the reflectance of printed samples were performed using a spectral photometer. Due to the use of black and white master batch material, the filaments also display differences in their color, and a high reflectance should correspond to a low rPLA content. Figure 12 shows the results for the printed samples. For the monoextruded filaments, the virgin material displayed a reflectance of 75%, while monoextruded filaments with 20%, 40% and 60% rPLA content had a decreased reflectance of 55%, 26% and 29%, respectively. On the contrary, the coextruded filament with a 20% skin layer of virgin material had a reflectance of 42%. All differences observed were statistically significant, with $p<1\cdot 10^{-8}$ for all comparisons but 40% rPLA v. 60% rPLA where p=0.046. This suggests that the skin layer was largely contributing to the overall reflectance of the samples.

## 4. Discussion

For the samples manufactured in the horizontal position, where the majority of the tensile strength comes from the strength of the individual strands, a recycled PLA content did not significantly affect the tensile strength. The samples manufactured in the vertical position, where the tensile strength is largely affected by the strength of the weld lines between the layers, there was a tensile strength comparable to the virgin and reference samples only for up to 40% recycled PLA content. The samples printed with 60% rPLA content had so much variation between the individual specimens, and no reliable conclusion can be reached. The impact strength was observed to decrease with increasing rPLA content, although the variation in the results does not allow for further conclusions on this matter.

While previous research regarding the mechanical properties of additively manufactured parts using recycled PLA focused on the degradation caused by the re-extrusion process [13,17], the material used in this study also experienced degradation through aging. This could serve as an explanation for why a larger decrease in tensile strength was observed. These results are in agreement with previous research by Ong et al. [31], where a 50% reduction in tensile strength after one recycling step was found, and after two cycles, only few specimen were actually able to be analyzed. Due to their setup using post-consumer PLA from a university 3D printing laboratory, their material is assumed to be fairly similar to the recycled material present in this study.

With increasing rPLA content, more instabilities in the extrusion process were observed in our experiments, leading to increased variation in the filament diameter and eventually to potential clogging of the nozzle during the AM process, which is consistent with previous findings [16,18,31,32].

The tensile tests performed highlighted a number of apparent differences between the monoextruded and the coextruded filaments. While all tensile tests performed on the monoextruded samples manufactured in the horizontal position showed some degree of ductile behavior and were in a similar tensile strength range, the coextruded samples were brittle with a tensile strength up to 38.5% lower than the monoextruded counterparts. The strength of the weld lines also did not improve through the coextrusion process. The tensile strength of the coextruded samples printed in the vertical position was 67% lower than the baseline samples made from virgin PLA.

One reason for this might be detectable in the microscopic analysis of the coextruded filaments. The filaments produced using coextrusion had an inconsistent quality. It is apparent that the outer layer of the virgin material, which was supposed to improve the strength of the weld line, has large variations in thickness and is not continuous. This leads to inconsistencies in the roundness and diameter of the filament and subsequently to variations in weight. The subsequent effects on the surface quality of the filament and thus the quality of the weld lines might be the underlying cause for the consistently low tensile strength. The authors’ hypothesis of coextrusion leading to better mechanical properties could therefore not be proven.

On the other hand, the reflectance measurements suggest that the coextrusion method presented is a viable option to reduce the amount of master batch and colorants needed for filament production. It has been confirmed that due to the laminar flow in the 3D printer’s hot-end, the color of the outer layer of the filament has the largest influence on the color of the additively manufactured part. Subsequently, the coextrusion method as presented here could be a useful tool to save production cost in the long-term, provided that further research increases the quality of filament produced by means of PLA–rPLA coextrusion.

## 5. Conclusions

In this study, monoextruded filaments for FFF with varying recycled PLA content have been produced and tested. It was found that the strength of the strand itself, tested by means of samples printed in the horizontal position, did not decrease significantly. The strength of the weld lines between the layers in samples printed in the vertical position on the other hand decreased noticeably, up to the point where no reliable results could be evaluated at 60% rPLA content.

To test the hypothesis that recycled filament with an outer layer of virgin material is able to form stronger weld lines than just recycled material, a method for the coextrusion of filament with a high rPLA content core layer and a 20% outer virgin PLA layer was developed. Coextruded filament was produced, and tensile as well as Charpy test samples in both horizontal and vertical positions were manufactured via extrusion-based AM. The parts manufactured from coextruded filament were found to be more brittle than their monoextruded counterparts. Due to inconsistencies in the coextrusion process, no continuous weld line could be formed between the layers, leading to a decreased tensile strength. However, the coextruded filament enabled the reduction of master batch and colorants as the outer layer of the filament appears to be mainly responsible for the manufactured part’s coloring. Therefore, switching to a coextrusion line could provide economical savings on master batch material.

Additional research improving methods to increase the use of recycled material in FFF filament is necessary. Further improvements in the coextrusion process are needed to make this a viable method. An improvement in the optical results could be achieved by compounding of the material and master batch prior to extrusion or the use of additional mixing elements before the die. The irregularities in the skin layer might be resolved by increasing the throughput to avoid flow surges. To address the issue of inconsistent filament diameter, the main cause for decreased mechanical properties of the parts manufactured from coextruded material, a closed-loop material feed system can be implemented in the AM process. By continuously measuring and correcting the material throughput, the reliance on a precise filament diameter can be circumvented. In addition, the use of a gear pump can help to reduce the pulsatility in the extruder output.

Finally, the optical results encourage further research in the concept of coextrusion for the saving of master batch. To test this concept, additional coextrusion trials with differently colored virgin material skin and core layers are necessary to analyze the mechanical performance of the coextrusion samples independently from the recycled PLA content, provided that further research increases the quality of filaments produced by means of PLA–rPLA coextrusion.

## Figures and Tables

**Table 1:** Process parameters for the manufacturing of test specimens.

Process Parameter	Value	Unit
Perimeters	2	[-]
Top/bottom layers	3	[-]
Layer height	0.3	[mm]
Manufacturing speed	49	[mm/s]
Top/bottom infill pattern	Rectilinear	[-]
Top/bottom infill angle	45°	[-]
Interior infill pattern	Gyroid	[-]
Interior infill density	40%	[-]
Extrusion width	0.84	[mm]
Temperature nozzle	230	[°C]
Temperature heat bed	55	[°C]
Part cooling	100%	[-]

## Data Availability

Data available on request.

## Abbreviations

The following abbreviations are used in this manuscript:

PLA	Polylactide Acid
rPLA	Recycled PLA
FFF	Fused Filament Fabrication
AM	Additive Manufacturing
MFI	Melt Flow Index
PC	Polycarbonate
ABS	Acrylonitrile Butadiene Styrene
HSD	Honestly Significant Difference
DIC	Differential Interference Contrast
LSM	Laser Scanning Microscope
IKV	Institute for Plastics Processing
